# Intracellular Responses to Ionizing Radiation: From Initial Damage to Cell Fate and Intercellular Communication

**DOI:** 10.3390/biology15211967

**Published:** 2026-11-02

**Authors:** Song Mi Lee, Moon-Taek Park, Sung Dae Kim

**Affiliations:** 1 Laboratory of Biochemistry & Cancer Immunology, College of Veterinary Medicine, Kyungpook National University, Daegu 41566, Republic of Korea; sm79lee@knu.ac.kr; 2 Research Center, Dongnam Institute of Radiological & Medical Sciences, Busan 46033, Republic of Korea

**Keywords:** ionizing radiation, DNA damage response, oxidative stress, intracellular signaling, cell fate, intercellular signaling

## Abstract

Ionizing radiation induces biological effects through direct energy deposition in biomolecules and indirect damage mediated by water radiolysis and reactive oxygen species. This review synthesizes the intracellular and intercellular events that connect these initial reactions to cell-fate determination. Following irradiation, cells coordinate DNA damage recognition, checkpoint control, and repair with oxidative stress and inflammatory signaling. Together, these responses connect the initial molecular damage with the intracellular and intercellular events that follow. The balance among damage severity, repair capacity, cell-cycle stage, genetic background, and microenvironmental signals shapes outcomes including recovery, transient or permanent arrest, senescence, mitotic catastrophe, apoptosis, regulated necrosis, and autophagy-associated survival or death. Irradiated cells further influence surrounding tissues through cytokines, chemokines, senescence-associated secretory factors, damage-associated molecular patterns, and cyclic GMP–AMP synthase–stimulator of interferon genes signaling. These integrated responses determine both therapeutic effects and normal-tissue injury, supporting investigation across molecular, cellular, immune, and tissue levels.

## Simple Summary

Ionizing radiation is widely used in medicine and research, but its effects on cells extend far beyond the initial damage caused at the moment of exposure. This review explains the sequence of events that begins when radiation directly strikes cellular molecules or reacts with water to form highly reactive substances. These early events can damage genetic material, proteins, fats, and cell structures. Cells then activate systems that detect and repair damage, pause cell division, and coordinate stress responses. Depending on the amount of damage and the cell’s ability to recover, a cell may survive, stop dividing, age prematurely, or die through several pathways. Irradiated cells can also release signals that affect nearby cells, inflammation, and immune responses. Viewing these events as one connected process helps explain why radiation can both destroy tumors and injure normal tissue. A clearer understanding of these linked responses may support safer and more effective radiation treatment and improve assessment of biological effects after radiation exposure.

## 1. Introduction

Ionizing radiation (IR) refers to radiation having high energy to pass through matter and induce ionization by removing electrons from atoms or molecules. Ionizing radiation, mainly used in the fields of biology and medicine, includes photon radiation such as X-rays and gamma rays, as well as particulate radiation such as alpha particles, beta particles, protons, and heavy ions. In particular, X-rays and gamma rays are widely used in the fields of radiation-biology research and radiation therapy because they have high penetration power and can induce various biological changes in cells and tissues [1,2].

Although these radiation types share the ability to induce ionization, their biological effects may differ considerably depending on radiation quality, particularly linear energy transfer (LET). Conventional X-rays and γ-rays are classified as low-LET radiation, whereas α-particles and heavier charged ions generally exhibit higher LET. The mechanistic pathways discussed in this review are primarily based on findings established using conventional low-LET irradiation, while relevant distinctions associated with higher-LET radiation are highlighted where appropriate [3].

When ionizing radiation is exposed to cells, the radiation energy is either directly transferred to biomolecules such as DNA, or it produces indirect biological effects through radiolysis of water molecules, which are abundant within cells. Reactive species generated by direct energy delivery and radiation can cause various forms of DNA damage, including DNA base damage, single-strand breaks (SSBs), and double-strand breaks (DSBs) [1,2,4]. In particular, complex DNA damage, in which DSBs and various types of DNA damage coexist in a narrow region, is considered an important form of radiation damage that cells cannot easily repair [2]. In addition, reactive oxygen species (ROS) generated after irradiation can affect not only DNA but also proteins, lipids, and organelles [1,5]. Therefore, the cellular response to radiation should be understood not as limited to simple DNA damage, but as a complex cellular stress response occurring throughout the cell.

When DNA damage occurs due to radiation, the cell recognizes it and rapidly activates the DNA damage response (DDR) to repair it. In this process, ATM (ataxia-telangiectasia mutated), ATR (ATM and Rad3-related), and DNA-dependent protein kinase (DNA-PK) act as major signaling molecules and activate various downstream signaling cascades [2,4]. These responses lead to the formation of γ-H2AX, activation of checkpoint kinases, and p53-dependent signaling, thereby regulating DNA repair and the cell-cycle checkpoint. If the damage is recoverable, the cell temporarily halts the cell cycle and repairs the DNA; however, if the damage is severe or persistent, it may progress to apoptosis, senescence, or other forms of cell death [1,4].

Radiobiology research is important not only for providing the essential foundation for improving the effectiveness of radiation therapy, but also for understanding radiation-induced damage to normal tissues and the biological effects of various types of radiation exposure. From the perspective of cancer treatment, ionizing radiation is a representative therapeutic method that suppresses the proliferation of tumor cells or induces their death through DNA damage. However, even when exposed to the same radiation, cells can respond differently depending on their cell type, genetic background, DNA repair capacity, and the state of intracellular signaling [1,5,6]. Therefore, understanding the initial molecular changes that occur in cells after irradiation and the corresponding intracellular signaling network is important for elucidating the mechanisms of radiation sensitivity and radiation resistance.

In the past, the biological effects of radiation were mainly explained from the perspective of DNA damage and the resulting cell killing. However, it is becoming clear that, at present, the cellular response to radiation is no longer confined to a simple process of cell death. Various intracellular signaling pathways, including the DNA damage response and ROS production, as well as ATM/ATR, p53, mitogen-activated protein kinase (MAPK), phosphoinositide 3-kinase (PI3K)/AKT, and nuclear factor-κB (NF-κB), can become activated after radiation exposure [1,5,6,7]. These signaling pathways can influence not only cell fate determinants such as DNA repair, cell-cycle arrest, apoptosis, and senescence, but also cell survival and inflammatory responses. In particular, DNA damage can be transmitted through ATM-dependent signaling to cytoplasmic signaling pathways such as NF-κB and p38 MAPK [7].

Furthermore, cells exposed to radiation can interact with surrounding cells by producing or secreting cytokines, chemokines, growth factors, and various soluble mediators. In addition, through mechanisms such as gap-junction-mediated communication between cells, various biological responses can be induced even in cells that have not been directly exposed to radiation, and this phenomenon is described as an important feature of the radiation-induced bystander effect (RIBE) [5,8,9].

Therefore, the radiation response should be understood not merely as changes occurring within the investigated individual cell, but as an intercellular response extending into the adjacent cells and the tissue microenvironment [8,9]. This is also an important concept for understanding changes in the tumor microenvironment and immune and inflammatory responses after radiation therapy.

**Figure 1:**
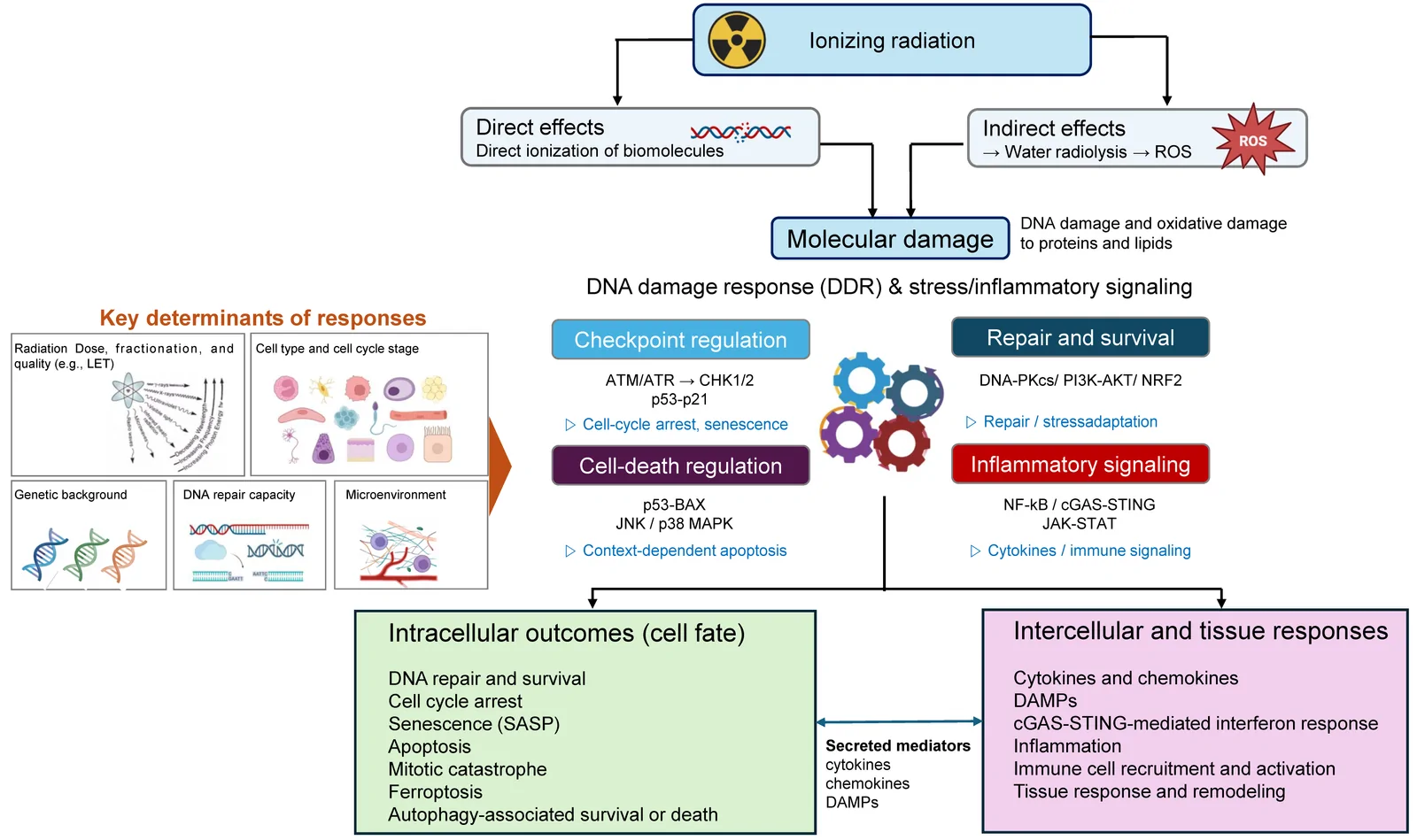
Overview of interconnected cellular responses to ionizing radiation. Ionizing radiation induces molecular damage through direct ionization of biomolecules and through the radiative degradation of water and the indirect action of reactive oxygen species (ROS). These DNA damage and oxidative stresses activate an interconnected signaling network involved in checkpoint regulation (ATM/ATR–CHK1/2 and p53–p21), DNA repair and survival (DNA-PKcs, PI3K–AKT, and NRF2), cell-death regulation (p53–BAX and JNK/p38 MAPK), and inflammatory signaling (NF-κB, cGAS–STING, and JAK–STAT). These signaling pathways regulate DNA repair and survival, cell cycle arrest, senescence, apoptosis, mitotic catastrophe, and other condition-dependent cellular responses through their interactions. At the same time, irradiated cells can affect surrounding cells and tissues through cytokines, chemokines, damage-associated molecular patterns (DAMPs), and immune-related signaling. The relative contribution of these interconnected signaling pathways varies depending on radiation dose and fractionation; radiation quality (e.g., LET) [10,11,12]; cell type and cell-cycle stage [13,14,15]; genetic background [1,16,17]; DNA repair capacity [13,18,19,20]; and the microenvironment [9,21,22].

This review aimed to present an integrated perspective by focusing on the temporal progression of cellular responses to radiation and their biological interrelations. In addition to intracellular events such as DNA damage, ROS generation, and cell cycle arrest caused by ionizing radiation, we further examine how these intracellular responses extend to intercellular communication and immune regulation through the release of cytokines, chemokines, and other signaling molecules. Importantly, these processes do not necessarily occur in a strictly linear sequence but involve overlapping pathways, feedback mechanisms, and context-dependent interactions. By considering the effects of radiation dose, radiation quality, cellular and genetic backgrounds, and the surrounding microenvironment, this review aims to provide a coherent conceptual framework for understanding how initial radiation-induced damage is translated into diverse cellular and tissue-level biological outcomes. This integrated framework is illustrated in Figure 1, while Table 1 summarizes representative molecular markers associated with radiation-induced DNA damage responses and repair, and Table 2 summarizes the approximate and overlapping temporal windows of representative radiation-induced cellular responses, emphasizing their biological interconnections and context-dependent progression. The relevant literature was primarily searched using PubMed with keywords such as ‘ionizing radiation’, ‘irradiation’, ‘DNA damage’, ‘DNA damage response’, ‘intracellular signaling’, ‘cell fate’, and ‘intercellular communication’, either individually or in combination. In addition, we reviewed the original articles that conducted the experiments directly before reviewing the review papers. However, review articles were also examined when necessary to provide a broad scientific context.

## 2. Physical and Chemical Changes in Cells Immediately After Radiation Exposure

The classical distinction between direct and indirect radiation action provides a fundamental basis for understanding the biological effects of ionizing radiation, as described in the pioneering radiobiological work of Lea [23]. Upon irradiation, energy is deposited in biological matter, leading to direct ionization or excitation of biomolecules and indirect chemical effects primarily mediated by water radiolysis [2,24]. Since most cells are composed of water, in biological damage caused by low-LET radiation, the indirect effect of water radiolysis plays an important role [24,25].

In direct action, the energy of the ionizing radiation is directly transferred to DNA, causing ionization or excitation of DNA molecules. During this process, various forms of DNA damage may occur, including DNA base modification, sugar–phosphate backbone damage, single-strand break (SSB), double-strand break (DSB), and DNA–protein cross-linking [2,24,26]. Clustered or complex DNA damage, in which multiple types of damage occur simultaneously in closely adjacent locations due to radiation, is difficult to repair and can have important effects on cell survival [2,26,27]. Importantly, the complexity and spatial distribution of radiation-induced DNA lesions are influenced by radiation quality and LET. Compared with low-LET radiation, high-LET radiation generally produces more densely clustered ionization events, resulting in a greater proportion of complex DNA lesions that can be difficult to repair accurately. Although clustered DNA damage can also occur following low-LET irradiation, its frequency and complexity tend to increase with LET [10].

In contrast, in indirect action, radiation energy is absorbed by water molecules, leading to water radiolysis. During this process, water molecules undergo ionization or excitation, resulting in the formation of highly reactive chemical species, including hydroxyl radicals (•OH), hydrated electrons (e^−^_a_q), and hydrogen radicals (H•). Subsequent chemical reactions involving these primary products can generate additional reactive oxygen species (ROS), such as superoxide anions (O_2_•^−^) and hydrogen peroxide (H_2_O_2_) [25,28]. Among these species, •OH is particularly reactive and readily interacts with surrounding biomolecules, making it a major contributor to indirect radiation-induced biological damage [24,25]. Classical radiation chemistry studies established the fundamental mechanisms of radical formation during water radiolysis. In particular, Draganic and Draganic investigated the primary yields of hydroxyl radicals and hydrated electrons during γ-radiolysis of water [29].

Therefore, if we simplify the initial process immediately after irradiation, it can be represented as the flow of ionizing radiation → water radiolysis → ROS generation → oxidative stress. However, the increase in ROS is not limited to DNA damage alone. ROS generated by radiation can not only damage the DNA bases and the sugar-phosphate backbone, but also oxidize the lipids of the cell membrane and organelles, and can induce protein amino acid side-chain oxidation, fragmentation, and structural changes [25,30]. Therefore, it is appropriate to understand the initial oxidative damage caused by radiation as a process that simultaneously affects biomolecules throughout the cell, including DNA, lipids, and proteins.

In particular, DNA damage caused by ROS is important for understanding the indirect effects of radiation. •OH generated through water radiolysis can react with DNA bases and deoxyribose to induce base modification and SSB; if complex damage occurs at adjacent sites, it can lead to DSB or clustered DNA damage [24,26,31]. These early DNA lesions subsequently activate a DNA damage response centered on ATM, ATR, and DNA-PK, and are linked to downstream signaling that determines cell cycle arrest, DNA repair, or cell death [2,4]. In other words, the cell’s response to radiation exposure can be seen as a continuous process that leads to physical energy deposition → the formation of reactive species through direct ionization of biomolecules or water radiolysis → damage to DNA, proteins, and lipids → activation of cellular signaling. These early physical and chemical changes become the starting point for subsequent DNA damage response, oxidative stress response, and intracellular signaling.

## 3. DNA Damage Response (DDR)

Ionizing radiation causes various types of damage to DNA, including base damage, single-strand breaks (SSB), double-strand breaks (DSB), and clustered DNA damage. Among these, DSB is considered one of the most important radiation-induced cytotoxic DNA lesions because it directly disrupts chromosome continuity and, if not repaired or repaired incorrectly, can lead to chromosome aberration, genomic instability, and cell death [18,19].

When DNA damage occurs due to radiation, the cell detects it and activates the DNA damage response (DDR) to repair the damage and regulate the fate of the cell. At the core of DDR are ATM, ATR, and DNA-PKcs, which belong to the phosphatidylinositol 3-kinase-related kinase (PIKK) family [4,20].

While ATM is mainly activated in response to DSBs, ATR plays a particularly important role in replication-associated DNA structures such as replication protein A (RPA)-coated single-stranded DNA formed during DNA end resection or stalled replication forks. DNA-PKcs, together with Ku70/Ku80, is a key factor that recognizes DNA ends and initiates non-homologous end joining (NHEJ) [20,32,33,34].

When DSB occurs due to radiation, the Ser139 residues of the histone variant H2AX are rapidly phosphorylated around the damaged site, leading to the formation of γ-H2AX [35]. As it spreads into the broad chromatin region around the DNA lesion site, it forms a platform through which DNA repair and signaling proteins can assemble at the lesion site. In 1998, Rogakou et al. first demonstrated that DNA double-strand breaks induce phosphorylation of H2AX at Ser139 [35], and it was later reported that ATM is the major kinase that phosphorylates H2AX after IR-induced DSB [36]. Therefore, γ-H2AX is now widely used as a representative experimental marker to evaluate radiation-induced DSB and DDR activation.

Activation of ATM and ATR leads to the activation of downstream checkpoint kinases, including CHK2 and CHK1, and ultimately regulates the function of p53 and various cell-cycle regulatory proteins [20,33,37]. Experimental studies have further demonstrated that radiation-induced ATR activation involves ATM/Mre11-dependent signaling and ATR autophosphorylation at Thr1989 [38,39]. Similarly, ATM-mediated phosphorylation of CHK2 at Thr68 has been demonstrated following ionizing radiation, with phosphorylated CHK2 accumulating at DNA damage sites [13]. This checkpoint signaling regulates the G1/S, intra-S, and G2/M checkpoints to prevent damaged DNA from being replicated or passed on to daughter cells. If DNA damage can be repaired, the cell temporarily halts the cell cycle and activates the repair pathway; however, if the damage is severe or persists, it may progress to apoptosis, senescence, or other forms of cell death [20].

The repair of radiation-induced DSB mainly utilizes two pathways: non-homologous end joining (NHEJ) and homologous recombination (HR) [18,19]. NHEJ operates during most cell-cycle phases by directly linking the ends of DNA, and it is an important DSB repair pathway in G1. When the Ku70/Ku80 heterodimer recognizes a damaged DNA end, DNA-PKcs binds, and then Artemis, XRCC4, XLF, and DNA ligase IV participate to process and join the DNA end [34,40]. Experimental evidence further indicates that cAMP–PKA signaling can modulate radiation-induced DNA-PKcs phosphorylation at Ser2056 and Thr2609 and impair NHEJ repair by reducing the recruitment of XRCC4 and DNA ligase IV to DNA damage sites in lung cancer cells [41]. Similarly, pharmacological inhibition of DNA-PKcs by BR101801 suppressed radiation-induced DNA-PKcs phosphorylation at Ser2056 without substantially altering Ku70/Ku80 protein levels, while delaying DNA damage repair and enhancing radiosensitivity in human cancer cells [42].

In contrast, HR uses homologous DNA sequences as templates to repair DSBs, so it generally has high accuracy and is mainly activated in the S and G2 phases, where sister chromatids can be utilized [14,43]. As part of the DSB is resected, single-stranded DNA is formed, and subsequently the damaged DNA sequence is restored through strand invasion and DNA synthesis using the homologous sequence [44]. BRCA2 plays a critical role in HR by facilitating RAD51 loading onto single-stranded DNA, thereby promoting the formation of RAD51 nucleoprotein filaments required for homologous strand invasion [45]. Experimental evidence further indicates that the subcellular localization of BRCA1 influences both DNA repair and cellular responses to irradiation, with nuclear BRCA1 supporting DSB repair and cytosolic accumulation contributing to enhanced radiation-induced cytotoxicity [46]. Therefore, the state of the cell cycle and the pattern of DNA damage become important factors in determining which repair pathway, NHEJ or HR, is selected [14,47].

In conclusion, DDR to ionizing radiation is not merely a simple DNA repair process, but an integrated cellular response that leads to DNA damage sensing → signal amplification → activation of the cell-cycle checkpoint → DNA repair or determination of cell fate. In particular, the ATM/ATR/DNA-PKcs–γ-H2AX–CHK1/CHK2–p53 axis provides a key signaling framework for understanding the early cellular response following radiation exposure [4,20]. However, it is more appropriate to understand this pathway not merely as a simple linear cascade, but as a network in which different signaling pathways overlap and branch off depending on the form of DNA damage, the cell cycle, and the cellular state. Representative molecular markers associated with radiation-induced DDR and DNA repair are summarized in Table 1.

**Table 1:** Representative molecular markers associated with DNA damage response and repair following ionizing radiation. Ionizing radiation induces DNA double-strand breaks (DSBs), leading to activation of the DNA damage response (DDR), DNA repair pathways, and cell-cycle checkpoints. Representative markers include γ-H2AX for DSB formation; phosphorylated ATM/CHK2 and ATR/CHK1 for DDR signaling; DNA-PKcs, Ku70/Ku80, and XRCC4 for non-homologous end joining (NHEJ); RAD51, BRCA1, and BRCA2 for homologous recombination (HR); and CHK1, CHK2, p53, and p21 for cell-cycle checkpoint activation.

Cellular Event/Pathway	Significance	Representative Markers	Experimental Model	Irradiation Conditions	Time After Irradiation	References
DNA double-strand break	DSB-associated chromatin response	γ-H2AX	SF268 cells	^137^Cs γ-rays, 1.2–10.8 Gy	30 min	[35]
DDR activation	ATM/ATR-dependent checkpoint signaling	p-ATM (Ser1981)	Human primary fibroblasts	IR, 0.5 Gy	5 min	[32]
		p-CHK2 (Thr68)	HeLa cells	IR, 5 Gy	1 h	[13]
		p-ATR (Thr1989)	HCT116 cells	IR, 10 Gy	2 h	[39]
		p-CHK1 (Ser317)	HeLa cells	IR, 5 Gy	1 h	[38]
NHEJ	DSB end joining	DNA-PKcs (Ser2056)	H1299 cells	γ-rays, 5 Gy	1 h	[41]
		Ku70/Ku80 (Ku86)	HCT116, H460 cells	IR, 2 Gy	2 h	[42]
		XRCC4	H1299, A549 cells	γ-rays, 5 Gy	1 h	[41]
HR	Homology-dependent DSB repair	RAD51	HSF1 human primary fibroblasts	X-rays, 2 Gy	2 h	[47]
		BRCA1	MCF7 cells	IR, 5 Gy	1 h	[46]
		BRCA2	U2OS cells	IR, 6 Gy	2 h	[45]
Cell-cycle checkpoint	Cell-cycle arrest	p-CHK1 (Ser317)	HeLa cells	IR, 5 Gy	1 h	[38]
		p-CHK2 (Thr68)	HeLa cells	IR, 5 Gy	1 h	[13]
		p53	MRC-5 cells	γ-rays, 2 Gy	8 h	[48]
		p21	MRC-5 cells	γ-rays, 2 Gy	8 h	[48]

## 4. Intracellular Signaling Activated by Ionizing Radiation

The cellular response to ionizing radiation goes beyond DNA damage and ROS production, leading to the activation of various intracellular signaling pathways. These signaling networks play an important role in determining whether, after radiation exposure, a cell will repair damage and survive, enter a temporary or permanent cell-cycle arrest, or proceed to cell death. In particular, pathways such as mitogen-activated protein kinase (MAPK), nuclear factor κB (NF-κB), and phosphoinositide 3-kinase (PI3K)–AKT are closely associated with stress responses caused by radiation [49,50,51].

The MAPK family typically includes ERK, JNK, and p38 MAPK. These kinases respond to various extracellular and intracellular stresses and can also be activated by ionizing radiation [21,49].

Generally, ERK signaling is closely associated with growth factor-mediated cell proliferation and survival through the Ras–Raf–MEK–ERK pathway [52,53], whereas JNK and p38 MAPK are prominent stress-responsive kinases activated by ROS, DNA damage, and other cellular stresses [54]. However, the functions of these pathways are not absolutely distinct, and they can have different effects on survival or cell death depending on the type of cell, the dose of radiation, and the time after irradiation [49,55].

Radiation can also activate the NF-κB signaling pathway. NF-κB is a representative transcription factor involved in inflammation, immune response, cell survival, and stress adaptation. When a DNA double-strand break occurs, ATM-dependent signaling can transmit the nuclear DNA damage signal to the cytoplasmic IKK complex, thereby inducing NF-κB activation, and ROS also acts as an important factor in regulating NF-κB signaling [7,50]. Therefore, NF-κB activation can be understood as one of the important signaling mechanisms that link radiation-induced DNA damage with inflammatory gene expression.

The PI3K–AKT pathway is also a representative pro-survival signaling pathway that can be activated by radiation. Ionizing radiation can activate PI3K and AKT through upstream signaling pathways such as receptor tyrosine kinases, and activated AKT can contribute to the cell’s resistance to radiation by inhibiting apoptosis and promoting cell survival and DNA damage repair [6,51,56]. In fact, Zingg and others experimentally reported that ionizing radiation can activate the PI3K/AKT survival pathway in tumor cells and vascular endothelial cells [51]. Furthermore, Toulany et al. demonstrated that AKT1 contributes to DNA-PKcs-dependent DNA double-strand break repair in human tumor cells, and that AKT1 inhibition enhances radiation-induced cytotoxicity by impairing this repair process [50].

The signaling response that appears after irradiation also includes a cytokine-dependent pathway. For example, IL-6, interferon, and other cytokines produced or secreted by radiation can bind to the corresponding receptor and activate JAK–STAT signaling [57,58]. Therefore, the JAK–STAT pathway can be regarded not as an early stress response directly activated by radiation, such as MAPK- or ATM-dependent signaling, but rather as an important secondary signaling pathway that links the radiation-altered extracellular environment with intracellular responses [11]. Because of these characteristics, it is useful to understand JAK–STAT signaling together with the post-radiation inflammatory response and cytokine/chemokine signaling.

Meanwhile, ROS increased by ionizing radiation does not act merely as a simple cytotoxic molecule, but activates a redox-sensitive signaling pathway. Notably, nuclear factor erythroid 2-related factor 2 (NRF2) is a key transcription factor that regulates the cell’s defensive response to oxidative stress [59]. Under normal conditions, NRF2 is regulated for degradation by KEAP1, but when oxidative stress increases, NRF2 becomes stabilized, migrates to the nucleus, and binds to the antioxidant response element (ARE) [59,60]. As a result, expression of various antioxidant and cytoprotective genes, including heme oxygenase-1 (HO-1), NAD(P)H quinone oxidoreductase 1 (NQO1), and glutathione-related enzymes, increases [16,61].

It has also been experimentally reported that ionizing radiation activates the NRF2–ARE pathway. McDonald and colleagues observed that after irradiation, the NRF2-dependent antioxidant response was activated and the expression of HO-1 and others increased, suggesting that this response may contribute to the cell’s adaptation to and survival from radiation [61]. Therefore, the NRF2–HO-1 axis can be regarded as a representative signaling pathway that links radiation-induced oxidative stress with the cell’s antioxidant defense.

In summary, ionizing radiation does not act merely as a stimulus that unilaterally induces cell death, but simultaneously activates multiple stress-response signaling networks through DNA damage and oxidative stress. The MAPK, NF-κB, and PI3K–AKT pathways regulate cell survival, inflammation, and cell fate, while the NRF2–HO-1 pathway induces antioxidant defense in response to radiation-induced oxidative stress. In addition, this intracellular response can further expand into interactions with surrounding cells and inflammatory reactions by activating JAK–STAT signaling through cytokines secreted after irradiation.

## 5. Determining the Fate of Cells Exposed to Radiation

Cells that are damaged by ionizing radiation exposure do not necessarily undergo cell death. Cell death is determined not only by the degree of initial DNA damage to the cell but also by the progress of the DNA repair process and the integrity of the cell cycle gate signaling system. Importantly, experimental evidence indicates that the initial number of DNA double-strand breaks does not fully predict whether irradiated cells recover or permanently withdraw from the cell cycle. Feringa et al. demonstrated that persistent DNA repair intermediates during G2 arrest enhance ATR-dependent signaling and p21 accumulation, promoting permanent cell-cycle exit and senescence [62]. Similarly, prolonged p53-dependent G1 arrest accompanied by sustained p21 expression has been observed in irradiated normal human fibroblasts [63]. In contrast, dysregulation of G2/M checkpoint control can contribute to abnormal mitotic progression and mitotic catastrophe, as demonstrated in irradiated HeLa cells [64]. Radiation quality further modifies these responses by influencing the complexity and repairability of DNA lesions. High-linear energy transfer (LET) radiation generally produces more spatially clustered DNA damage than low-LET radiation, potentially increasing the persistence of unrepaired lesions and altering cellular radiosensitivity [22,65]. Together, these findings indicate that radiation-induced cell fate is shaped not simply by the initial damage burden, but by the interplay among DNA repair progression, checkpoint regulation, and damage complexity.

Puck et al. established an experimental basis for quantifying the radiation response of mammalian cells in terms of survival after X-ray irradiation, based on their colony-forming ability [48]. This pioneering study established clonogenic survival as a fundamental measure of cellular radiosensitivity, reflecting the ability of irradiated cells to retain reproductive capacity rather than merely survive the immediate effects of radiation. Subsequently, Elkind and Sutton (1959) provided experimental evidence that mammalian cells can recover from sublethal radiation damage [17]. These studies demonstrated that when assessing a cell’s sensitivity to radiation, one must consider not only immediate intracellular damage but also the cell’s ability to recover and maintain long-term proliferative capacity; to this day, clonogenic assays remain the gold standard for evaluating cellular response to radiation despite remarkable advances in life sciences.

Following irradiation, DNA damage activates checkpoint signaling pathways involving ATM–CHK2, ATR–CHK1, and p53–p21, which coordinate cell-cycle arrest with DNA repair [32,37,63]. Consistent with this response, increased levels of phosphorylated p53 (Ser15), total p53, and p21 have been observed in normal human fibroblasts following γ-irradiation [66]. Although transient checkpoint activation can provide time for DNA repair and subsequent cell-cycle re-entry, persistent damage signaling may promote prolonged or permanent growth arrest [62,63]. Importantly, Mirzayans et al. demonstrated that p21 plays a critical role in radiation-induced accelerated senescence in certain TP53 wild-type human tumor cell lines, supported by experiments using CDKN1A-deficient cells [67]. These findings indicate that radiation-induced senescence is influenced not only by the persistence of DNA damage signaling but also by the functional status of cell-cycle checkpoint regulators, particularly p21.

Apoptosis is also one of the well-known forms of ionizing radiation-induced regulated cell death. Lowe et al. demonstrated that p53 is required for efficient radiation-induced apoptosis in mouse thymocytes [68]. However, Strasser et al. showed that proliferating lymphoid cells lacking p53 could still undergo apoptosis following irradiation, whereas nonproliferating lymphocytes were relatively resistant. Furthermore, Bcl-2 expression suppressed apoptosis and increased clonogenic survival in these cells [69]. These findings indicate that apoptotic sensitivity depends not only on p53 status but also on cellular proliferative state and anti-apoptotic signaling. At the mechanistic level, mitochondrial involvement in radiation-induced apoptosis has been demonstrated through cytochrome c release and Apaf-1/caspase-9-dependent signaling in a cell-free experimental system [70]. Thus, although apoptosis is a well-established radiation-induced cell death mechanism, its relative contribution to cellular radiosensitivity varies according to cell type, genetic background, and survival signaling.

Radiation-induced cellular senescence is characterized by stable proliferative arrest despite continued cellular viability and metabolic activity [67,71]. Although this response limits the clonogenic potential of irradiated tumor cells, senescent cells are not necessarily eliminated and may exert biological effects on surrounding cells. Importantly, senescent cells can develop a senescence-associated secretory phenotype (SASP), characterized by the secretion of inflammatory cytokines, chemokines, and other extracellular mediators [72]. Rodier et al. demonstrated that persistent DNA damage signaling, rather than transient DDR activation, promotes delayed secretion of inflammatory cytokines, including IL-6 and IL-8, following irradiation [73]. Notably, this response required ATM, NBS1, and CHK2 signaling but was not dependent on the cell-cycle arrest regulators p53 and pRb [73]. These findings indicate that senescence-associated growth arrest and inflammatory secretory responses, although frequently linked, are not necessarily regulated by identical molecular mechanisms. Thus, radiation-induced senescence may simultaneously suppress cellular proliferation and promote intercellular communication through persistent DNA damage-associated signaling and SASP.

In addition to apoptosis and senescence, mitotic catastrophe is a well-established cellular response to irradiation that can occur when cells undergo aberrant mitosis with unresolved DNA damage or defective checkpoint regulation. Rather than representing a distinct cell death execution pathway, mitotic catastrophe is generally regarded as a process associated with abnormal mitotic progression that may ultimately lead to cell death or permanent proliferative arrest. Ianzini and Mackey demonstrated that X-irradiated HeLa S3 cells exhibited cyclin B1 accumulation, premature chromosome condensation, and nuclear fragmentation following prolonged cell-cycle delays [64]. Similarly, Leskovac et al. observed persistent DNA damage and abnormal nuclear morphologies, including micronuclei and multinucleation, in irradiated FANCD2-deficient primary fibroblasts [12]. These findings suggest that DNA repair capacity and checkpoint regulation influence whether irradiated cells undergo abnormal mitotic progression. Thus, although mitotic catastrophe is a well-recognized consequence of radiation-induced mitotic dysfunction, its occurrence and contribution to the loss of proliferative capacity depend on the cellular context and the functional integrity of DNA repair and checkpoint pathways.

Beyond the major cellular outcomes described above, additional forms of regulated cell death, including necroptosis, pyroptosis, and ferroptosis, have been identified following irradiation in specific experimental models. Radiation-induced necroptosis has been linked to RIPK1–RIPK3–MLKL signaling, with experimental evidence indicating that necroptotic tumor cells can promote the proliferation of surviving cells through JNK–IL-8 signaling [74]. Similarly, radiation-induced pyroptosis has been demonstrated in GSDME-expressing tumor cells through caspase-9–caspase-3–GSDME activation, with potential consequences for antitumor immune responses [75]. More extensive mechanistic evidence has also established a contribution of ferroptosis, an iron-dependent form of cell death driven by lipid peroxidation, to radiation-induced tumor cell killing. Ye et al. demonstrated that radiation-induced lipid peroxidation contributes to ferroptotic cell death and that ferroptosis-inducing agents can enhance the antitumor effects of irradiation [76]. Furthermore, Lei et al. showed that radiation-induced ACSL4 expression promotes lipid peroxidation and ferroptosis, whereas SLC7A11 and GPX4 counteract this response and contribute to radioresistance [15]. Collectively, these studies demonstrate that alternative regulated cell death pathways can contribute to radiation responses, but their activation and biological significance depend on the availability of specific molecular regulators and the cellular and experimental context. They should therefore be distinguished from the more broadly established DNA damage and checkpoint responses to irradiation.

Autophagy is another well-known cellular response induced by ionizing radiation, but the induction of autophagy does not necessarily mean cell death. Paglin et al. observed that irradiation induces the formation of acidic vesicular organelles associated with autophagy in human cancer cells, and that inhibition of vesicular acidification increased clonogenic cell death, suggesting a potential cytoprotective role for this response [77]. Furthermore, Apel et al. showed that suppression of autophagy-related genes, including BECN1 and ATG5, enhanced radiosensitivity in several radioresistant carcinoma cell lines, although the effects differed among cellular models [78]. These findings indicate that radiation-induced autophagy can function as an adaptive survival mechanism, but its contribution to cellular radiosensitivity is context-dependent. Therefore, autophagy induction should be distinguished from autophagy-dependent cell death, which requires direct evidence that autophagic machinery contributes to cell death rather than merely accompanying the radiation response.

Radiation-induced tumor cell death can also promote antitumor immune responses through immunogenic cell death (ICD), although this response is not universally observed. Several experimental studies have identified cell-surface calreticulin (CRT) exposure, extracellular ATP release, and high-mobility group box 1 (HMGB1) release as representative indicators of ICD [79,80]. These molecules contribute to distinct immunological processes, including the recognition and uptake of dying tumor cells by antigen-presenting cells, immune cell recruitment, and antigen presentation. Importantly, Obeid et al. demonstrated that CRT exposure is required for the immunogenicity of γ-irradiation-induced apoptosis in the experimental models examined, providing direct evidence for the functional importance of this marker [81]. Similarly, Apetoh et al. showed that HMGB1 released from dying tumor cells promotes antigen processing and presentation through TLR4-dependent signaling in dendritic cells [79]. Golden et al. further demonstrated that irradiation induces ICD-associated signals, including CRT exposure and ATP and HMGB1 release, in a dose-dependent manner [80]. Nevertheless, the detection of these molecular indicators alone does not establish functional ICD. Demonstration of protective antitumor immunity, for example through vaccination assays using dying tumor cells in immunocompetent hosts, provides stronger functional evidence. Thus, ICD-associated molecular markers are valuable for evaluating the immunogenic potential of irradiated cells, but their biological significance must be interpreted together with functional immune responses.

Taken together, these findings demonstrate that radiation-induced cell fate cannot be explained by a simple distinction between survival and cell death. Rather, cellular outcomes emerge from the interplay among DNA damage persistence, repair capacity, checkpoint integrity, genetic background, and stress-responsive signaling. Importantly, loss of clonogenic capacity does not necessarily indicate immediate cell death, as irradiated cells may undergo prolonged growth arrest or senescence while remaining metabolically active. Conversely, different forms of cell death can occur through different molecular mechanisms, and the extent to which these mechanisms contribute varies depending on the cellular and experimental context. These intracellular responses can also affect the surrounding environment by releasing cytokines, chemokines, age-related secretory factors, and damage-associated molecular patterns. Such intercellular signaling may occur concurrently with, rather than strictly after, cell-fate determination. Therefore, radiation-induced cellular responses should be understood as interconnected and potentially overlapping processes that extend from intracellular damage responses to tissue-level biological consequences. The approximate temporal relationships among these events and their representative molecular indicators are summarized in Table 2.

**Table 2:** Approximate temporal course and biological interrelations of radiation-induced cellular responses: major events, molecular markers, and experimental approaches. Ionizing radiation induces a series of interconnected biological responses, which unfold over multiple time scales and often overlap. The initial physical and chemical events occur within a very short time, followed by activation of DNA damage response, stress signal transduction, DNA repair, and cell cycle regulation. Subsequently, cellular consequences such as apoptosis, senescence, mitotic catastrophe, and changes in the secretion of cytokines and chemokines occur, and these effects can last from several hours to several days, or even longer. The important point is that these reactions do not necessarily occur strictly sequentially, and the temporal intervals in which each reaction takes place are often overlapped. The time range presented in the table is only an approximate figure and should not be interpreted as a universal standard. This is because the onset time, duration, and intensity of each reaction vary depending on the type of radiation, linear energy transfer (LET), dose, cell type, genetic background, and experimental conditions. Therefore, this table does not represent a fixed chronological order, but rather provides a conceptual framework for interpreting the temporal and biological interrelations between radiation-induced cellular events.

Time After Irradiation	Cellular Event	Representative Markers/Indicators	Representative Assays/Experiments	References
10^−15^–10^−6^ s	Ionization/water radiolysis	Hydroxyl radical (•OH), hydrated electron (e_a_q^−^), hydrogen radical (H•), hydrogen peroxide (H_2_O_2_)	Pulse radiolysis, EPR/ESR, chemical dosimetry	[29]
seconds–minutes (approx.)	ROS generation	ROS	DCFDA/MitoSOX assay	[61]
	DNA damage	DNA strand breaks	Gel electrophoresis	[27]
minutes–hours (approx.)	DDR activation	γ-H2AX, p-ATM, p-ATR, p-CHK1/2	Western blotting, immunofluorescence, flow cytometry	[13,32,38,39]
	Stress signaling	p-MAPK, NF-κB p65	Western blotting, reporter assay	[21,49,50]
hours–days (approx.)	DNA repair	RAD51, 53BP1, DNA-PKcs, Ku70/80	Repair-foci IF, Western blotting	[41,45,47]
	Cell-cycle arrest	p53, p21	Western blotting, RT-qPCR, cell-cycle analysis	[63,66]
	Cytokine/chemokine production	IL-1β, IL-6, IL-8, CXCL10	ELISA, multiplex assay, RT-qPCR	[57,73,82,83]
hours–days and later (approx.)	Apoptosis	Cleaved caspase-3/PARP, Annexin V	Western blotting, Annexin V/PI, TUNEL	[68,69,70]
	Senescence	p16, p21, SA-β-gal	SA-β-gal staining, Western blotting	[62,67]
	Mitotic catastrophe	Micronuclei, multinucleation	Microscopy	[12,64]
	Altered secretome	SASP factors	ELISA, multiplex assay, proteomic analysis	[72,73]

## 6. Signals Sent by Cells to the Surrounding Environment After Radiation Exposure

The cellular response to ionizing radiation does not end within irradiated cells. Radiation-induced DNA damage and oxidative stress can activate intracellular signaling pathways such as NF-κB and MAPK, while additional context-dependent pathways, including cGAS–STING and cytokine-mediated JAK–STAT signaling, may contribute to the production and release of cytokines, chemokines, and damage-associated molecular patterns (DAMPs) [5,82,84,85]. These extracellular mediators regulate local inflammatory and immune responses and ultimately shape tissue-level radiation responses by acting on adjacent tumor cells, stromal cells, and immune cells.

Representative soluble mediators that appear after irradiation may include cytokines and chemokines such as IL-1β, IL-6, IL-8, TNF-α, and CXCL10. However, the patterns of their expression and secretion can vary greatly depending on the type of cell, the radiation dose, the time after irradiation, and the state of cell activation. For example, radiation-induced inflammatory signaling can be regulated by the NF-κB and MAPK pathways, and it has also been reported that in macrophages, IL-1β production increases through inflammasome activation [86]. Therefore, the cytokine/chemokine response mediated by radiation should be understood not merely as a simple common response, but as a cell-specific stress-response program.

In certain cellular and experimental contexts, radiation-induced DNA damage can activate innate immune sensing pathways, including the cyclic GMP–AMP synthase–stimulator of interferon genes (cGAS–STING) pathway. Following irradiation, chromosome damage and micronucleus formation may result in the accumulation of cytosolic DNA, which can be recognized by cGAS [11,87]. Activated cGAS generates cyclic GMP–AMP (cGAMP), leading to STING activation and downstream TBK1–IRF3 signaling, which can induce type I interferon responses, including IFN-β production [85,88,89]. Although the molecular mechanisms of cGAS–STING signaling are well established, its activation following irradiation is not universal. The magnitude and biological consequences of this response depend on factors such as cell type, functional integrity of the pathway, radiation dose and fractionation, and the surrounding immune microenvironment. In particular, radiation-induced TREX1 expression under certain high-dose irradiation conditions may limit cytosolic DNA accumulation and attenuate cGAS–STING-dependent signaling [85]. Thus, cGAS–STING represents an important but context-dependent mechanistic link between radiation-induced DNA damage and innate immune responses.

Type I IFN signaling is not limited to a simple antiviral response but also plays an important role in radiation-induced antitumor immunity. IFN signaling can promote dendritic cell activation and antigen presentation, and can increase the expression of various chemokines, thereby influencing the recruitment of effector immune cells [83]. In particular, CXCR3 ligands such as CXCL9, CXCL10, and CXCL11 are associated with the migration of cytotoxic T lymphocytes and NK cells, and it has also been experimentally reported that STING signaling is required for radiation-induced CXCL10 and CCL5 expression [90]. Therefore, the sequence of DNA damage → cGAS–STING → type I IFN → chemokine production → immune-cell recruitment can be considered a representative mechanism by which damage inside cells after radiation exposure is amplified into the surrounding immune response.

The signals transmitted to the surrounding environment after irradiation include not only cytokines and chemokines, but also DAMPs. In particular, during the process that induces immunogenic cell death (ICD), exposure of calreticulin (CRT) on the cell surface, extracellular ATP release, and the release of high-mobility group box 1 (HMGB1) can appear as representative immunogenic signals [80,84]. CRT functions as an “eat-me” signal that promotes phagocytosis of dying cells, extracellular ATP can activate antigen-presenting cells through purinergic receptor signaling, and HMGB1 can influence innate immune signaling via pattern-recognition receptors [79,91]. Golden and colleagues experimentally demonstrated that in irradiated tumor cells, the exposure of the cell surface to calreticulin and the release of ATP and HMGB1 can increase depending on the radiation dose, experimentally suggesting that ionizing radiation can induce characteristic features of immunogenic cell death [80]. Therefore, radiation-induced cell death is not merely a process of removing damaged cells, but may also serve as a signaling event through which the dying cell conveys its status to the surrounding immune system.

However, the immune response induced by radiation does not always act in an anti-tumor direction. Radiation-induced cytokines and chemokines may promote the recruitment of effector immune cells, but depending on the situation, they may also increase chronic inflammation, immunosuppressive cell recruitment, or tissue injury. It has also been reported that at very high single-dose radiation doses, increased expression of the exonuclease TREX1, which degrades cytosolic DNA, may limit cGAS–STING-dependent immunogenic signaling [85]. Therefore, the final outcome of radiation-induced intercellular signaling may vary depending on the dose and fractionation, the cell type, and the tissue microenvironment.

In conclusion, the cell response to ionizing radiation can be understood as a continuous process that extends to DNA damage and ROS formation, → intracellular stress signaling, → cytokine/chemokine and DAMP release, and → responses of neighboring cells and immune cells. From this perspective, radiation acts not merely as a physical stimulus that kills irradiated cells, but as a biological stimulus that alters communication between cells and the tissue microenvironment.

## 7. Conclusions

The cellular response to ionizing radiation is not a single process that can be explained by simple DNA damage and cell death. Radiation energy directly ionizes biomolecules or generates ROS through water radiolysis, causing damage to DNA, proteins, and lipids. These early lesions activate the DNA damage response centered on ATM, ATR, and DNA-PKcs, as well as stress-response signaling pathways such as MAPK, NF-κB, PI3K–AKT, and NRF2. Subsequently, depending on the degree of damage and the cell’s ability to repair itself, as well as the cell cycle and the cell’s intrinsic characteristics, cells may exhibit different fates, including DNA repair and survival, transient or permanent cell-cycle arrest, senescence, mitotic catastrophe, and various forms of cell death.

Furthermore, the response to radiation is not confined solely to the interior of the irradiated cells. SASP produced by senescent cells, radiation-induced cytokines and chemokines, and DAMPs released by dying cells induce interactions with surrounding cells and immune cells. In particular, cGAS–STING–type I interferon signaling and immunogenic cell death are attracting attention as important mechanisms linking radiation-induced DNA damage with innate/adaptive immune responses. Therefore, the radiation response should be understood as a continuous and integrated biological response that leads to physical and chemical damage → intracellular signaling → cell fate determination → intercellular and tissue responses.

These cellular responses to radiation can vary greatly depending not only on the type and dose of radiation, but also on the cell type, genetic background, cell cycle, and the surrounding microenvironment. In the future, beyond independently analyzing individual signaling pathways, research that clarifies how these responses are connected over time and across space and influence one another will be important. This integrated understanding not only helps in understanding the fundamental principles of cellular response to radiation, but can also provide a foundation for developing new research strategies to enhance the effectiveness of radiation therapy and reduce damage to normal tissues.

An important consideration is that radiation-induced cellular responses are highly context-dependent. Even under similar irradiation conditions, cellular outcomes may differ according to radiation dose and quality, DNA repair capacity, genetic background, cell type, and the surrounding microenvironment. Despite substantial advances in understanding radiation-induced signaling, several important questions remain unresolved. Moreover, it remains unclear how the intensity and duration of DNA damage responses interact with cellular stress and survival signaling pathways to determine the fate of specific cells. Further research is needed on how radiation-induced senescence, regulated apoptosis, and inflammatory signaling affect long-term tissue responses.

Therefore, future research should elucidate the temporal and context-dependent interactions among DNA damage responses, intracellular signaling networks, and intercellular communication. These studies can help identify the key factors that determine sensitivity or resistance to radiation and enable researchers to predict cellular responses to irradiation more accurately. In particular, a deeper understanding of these mechanisms can be crucial for developing strategies that enhance cancer cells’ radiosensitivity while minimizing toxicity to normal tissues in the field of radiation therapy. From the perspective of radiation protection, identifying the molecular and cellular factors associated with radiation-induced tissue injury and delayed biological effects may improve the assessment of radiation risks and support the development of more effective protective strategies.

## Data Availability

No new data were created or analyzed in this study. Data sharing is not applicable to this article.

## References

[1] Kim B.M., Hong Y., Lee S., Liu P., Lim J.H., Lee Y.H., Lee T.H., Chang K.T., Hong Y. (2015). Therapeutic Implications for Overcoming Radiation Resistance in Cancer Therapy. Int. J. Mol. Sci..

[2] Wilkinson B., Hill M.A., Parsons J.L. (2023). The Cellular Response to Complex DNA Damage Induced by Ionising Radiation. Int. J. Mol. Sci..

[3] Paganetti H. (2018). Proton Relative Biological Effectiveness—Uncertainties and Opportunities. Int. J. Part. Ther..

[4] Zhang C., Liu J., Wu J., Ranjan K., Cui X., Wang X., Zhang D., Zhu S. (2024). Key molecular DNA damage responses of human cells to radiation. Front. Cell Dev. Biol..

[5] Valerie K., Yacoub A., Hagan M.P., Curiel D.T., Fisher P.B., Grant S., Dent P. (2007). Radiation-induced cell signaling: Inside-out and outside-in. Mol. Cancer Ther..

[6] Hein A.L., Ouellette M.M., Yan Y. (2014). Radiation-induced signaling pathways that promote cancer cell survival (review). Int. J. Oncol..

[7] Yang Y., Xia F., Hermance N., Mabb A., Simonson S., Morrissey S., Gandhi P., Munson M., Miyamoto S., Kelliher M.A. (2011). A cytosolic ATM/NEMO/RIP1 complex recruits TAK1 to mediate the NF-kappaB and p38 mitogen-activated protein kinase (MAPK)/MAPK-activated protein 2 responses to DNA damage. Mol. Cell. Biol..

[8] Nagasawa H., Little J.B. (1992). Induction of sister chromatid exchanges by extremely low doses of alpha-particles. Cancer Res..

[9] Azzam E.I., de Toledo S.M., Little J.B. (2001). Direct evidence for the participation of gap junction-mediated intercellular communication in the transmission of damage signals from alpha -particle irradiated to nonirradiated cells. Proc. Natl. Acad. Sci. USA.

[10] Hada M., Georgakilas A.G. (2008). Formation of Clustered DNA Damage after High-LET Irradiation: A Review. J. Radiat. Res..

[11] Harding S.M., Benci J.L., Irianto J., Discher D.E., Minn A.J., Greenberg R.A. (2017). Mitotic progression following DNA damage enables pattern recognition within micronuclei. Nature.

[12] Leskovac A., Petrovic S., Guc-Scekic M., Vujic D., Joksic G. (2014). Radiation-induced mitotic catastrophe in FANCD2 primary fibroblasts. Int. J. Radiat. Biol..

[13] Ward I.M., Wu X., Chen J. (2001). Threonine 68 of Chk2 is phosphorylated at sites of DNA strand breaks. J. Biol. Chem..

[14] Kai R., Ines K., Larry H.T., Markus L. (2003). Pathways of DNA Double-Strand Break Repair during the Mammalian Cell Cycle. Mol. Cell. Biol..

[15] Lei G., Zhang Y., Koppula P., Liu X., Zhang J., Lin S.H., Ajani J.A., Xiao Q., Liao Z., Wang H. (2020). The role of ferroptosis in ionizing radiation-induced cell death and tumor suppression. Cell Res..

[16] Thimmulappa R.K., Mai K.H., Srisuma S., Kensler T.W., Yamamoto M., Biswal S. (2002). Identification of Nrf2-regulated genes induced by the chemopreventive agent sulforaphane by oligonucleotide microarray. Cancer Res..

[17] Elkind M.M., Sutton H. (1959). X-ray damage and recovery in mammalian cells in culture. Nature.

[18] Kavanagh J.N., Redmond K.M., Schettino G., Prise K.M. (2013). DNA double strand break repair: A radiation perspective. Antioxid. Redox Signal..

[19] Mahaney B.L., Meek K., Lees-Miller S.P. (2009). Repair of ionizing radiation-induced DNA double-strand breaks by non-homologous end-joining. Biochem. J..

[20] Blackford A.N., Jackson S.P. (2017). ATM, ATR, and DNA-PK: The Trinity at the Heart of the DNA Damage Response. Mol. Cell.

[21] Chen Y.R., Meyer C.F., Tan T.H. (1996). Persistent activation of c-Jun N-terminal kinase 1 (JNK1) in gamma radiation-induced apoptosis. J. Biol. Chem..

[22] Nikitaki Z., Nikolov V., Mavragani I.V., Mladenov E., Mangelis A., Laskaratou D.A., Fragkoulis G.I., Hellweg C.E., Martin O.A., Emfietzoglou D. (2016). Measurement of complex DNA damage induction and repair in human cellular systems after exposure to ionizing radiations of varying linear energy transfer (LET). Free Radic. Res..

[23] Lea D.E. (1946). Actions of Radiations on Living Cells.

[24] von Sonntag C. (1991). The chemistry of free-radical-mediated DNA damage. Basic. Life Sci..

[25] Azzam E.I., Jay-Gerin J.P., Pain D. (2012). Ionizing radiation-induced metabolic oxidative stress and prolonged cell injury. Cancer Lett..

[26] Cadet J., Ravanat J.L., TavernaPorro M., Menoni H., Angelov D. (2012). Oxidatively generated complex DNA damage: Tandem and clustered lesions. Cancer Lett..

[27] Sutherland B.M., Bennett P.V., Sidorkina O., Laval J. (2000). Clustered DNA damages induced in isolated DNA and in human cells by low doses of ionizing radiation. Proc. Natl. Acad. Sci. USA.

[28] Reisz J.A., Bansal N., Qian J., Zhao W., Furdui C.M. (2014). Effects of ionizing radiation on biological molecules--mechanisms of damage and emerging methods of detection. Antioxid. Redox Signal..

[29] Draganic Z.D., Draganic I.G. (1973). Formation of primary yields of hydroxyl radical and hydrated electron in the .gamma.-radiolysis of water. J. Phys. Chem..

[30] Hawkins C.L., Davies M.J. (2019). Detection, identification, and quantification of oxidative protein modifications. J. Biol. Chem..

[31] Spotheim-Maurizot M., Davídková M. (2011). Radiation damage to DNA in DNA-protein complexes. Mutat. Res..

[32] Bakkenist C.J., Kastan M.B. (2003). DNA damage activates ATM through intermolecular autophosphorylation and dimer dissociation. Nature.

[33] Zou L., Elledge S.J. (2003). Sensing DNA Damage Through ATRIP Recognition of RPA-ssDNA Complexes. Science.

[34] Gottlieb T.M., Jackson S.P. (1993). The DNA-dependent protein kinase: Requirement for DNA ends and association with Ku antigen. Cell.

[35] Rogakou E.P., Pilch D.R., Orr A.H., Ivanova V.S., Bonner W.M. (1998). DNA double-stranded breaks induce histone H2AX phosphorylation on serine 139. J. Biol. Chem..

[36] Burma S., Chen B.P., Murphy M., Kurimasa A., Chen D.J. (2001). ATM phosphorylates histone H2AX in response to DNA double-strand breaks. J. Biol. Chem..

[37] Matsuoka S., Huang M., Elledge S.J. (1998). Linkage of ATM to Cell Cycle Regulation by the Chk2 Protein Kinase. Science.

[38] Myers J.S., Cortez D. (2006). Rapid activation of ATR by ionizing radiation requires ATM and Mre11. J. Biol. Chem..

[39] Liu S., Shiotani B., Lahiri M., Maréchal A., Tse A., Leung C.C., Glover J.N., Yang X.H., Zou L. (2011). ATR autophosphorylation as a molecular switch for checkpoint activation. Mol. Cell.

[40] Silvia C., Lisa W., Lucia A., Penny A.J., Alessandro V. (2007). Interaction of the Ku heterodimer with the DNA ligase IV/Xrcc4 complex and its regulation by DNA-PK. DNA Repair..

[41] Noh S.E., Juhnn Y.S. (2020). Inhibition of non-homologous end joining of gamma ray-induced DNA double-strand breaks by cAMP signaling in lung cancer cells. Sci. Rep..

[42] Lee J.H., Jeon B., Park M., Ha J., Kim S.J., Son M.K., Wang S., Lee J.H., Jeong Y.K. (2021). Synergistic radiosensitizing effect of BR101801, a specific DNA-dependent protein kinase inhibitor, in various human solid cancer cells and xenografts. Am. J. Cancer Res..

[43] Johnson R.D., Jasin M. (2000). Sister chromatid gene conversion is a prominent double-strand break repair pathway in mammalian cells. EMBO J..

[44] Sartori A.A., Lukas C., Coates J., Mistrik M., Fu S., Bartek J., Baer R., Lukas J., Jackson S.P. (2007). Human CtIP promotes DNA end resection. Nature.

[45] Sánchez H., Paul M.W., Grosbart M., van Rossum-Fikkert S.E., Lebbink J.H.G., Kanaar R., Houtsmuller A.B., Wyman C. (2017). Architectural plasticity of human BRCA2-RAD51 complexes in DNA break repair. Nucleic Acids Res..

[46] Wang H., Yang E.S., Jiang J., Nowsheen S., Xia F. (2010). DNA damage-induced cytotoxicity is dissociated from BRCA1′s DNA repair function but is dependent on its cytosolic accumulation. Cancer Res..

[47] Shibata A., Conrad S., Birraux J., Geuting V., Barton O., Ismail A., Kakarougkas A., Meek K., Taucher-Scholz G., Löbrich M. (2011). Factors determining DNA double-strand break repair pathway choice in G2 phase. EMBO J..

[48] Puck T.T., Marcus P.I. (1956). Action of x-rays on mammalian cells. J. Exp. Med..

[49] Carter S., Auer K.L., Reardon D.B., Birrer M., Fisher P.B., Valerie K., Schmidt-Ullrich R., Mikkelsen R., Dent P. (1998). Inhibition of the mitogen activated protein (MAP) kinase cascade potentiates cell killing by low dose ionizing radiation in A431 human squamous carcinoma cells. Oncogene.

[50] Li N., Karin M. (1998). Ionizing radiation and short wavelength UV activate NF-kappaB through two distinct mechanisms. Proc. Natl. Acad. Sci. USA.

[51] Zingg D., Riesterer O., Fabbro D., Glanzmann C., Bodis S., Pruschy M. (2004). Differential activation of the phosphatidylinositol 3′-kinase/Akt survival pathway by ionizing radiation in tumor and primary endothelial cells. Cancer Res..

[52] Moodie S.A., Willumsen B.M., Weber M.J., Wolfman A. (1993). Complexes of Ras.GTP with Raf-1 and mitogen-activated protein kinase kinase. Science.

[53] Erhardt P., Schremser E.J., Cooper G.M. (1999). B-Raf inhibits programmed cell death downstream of cytochrome c release from mitochondria by activating the MEK/Erk pathway. Mol. Cell. Biol..

[54] Benhar M., Dalyot I., Engelberg D., Levitzki A. (2001). Enhanced ROS production in oncogenically transformed cells potentiates c-Jun N-terminal kinase and p38 mitogen-activated protein kinase activation and sensitization to genotoxic stress. Mol. Cell. Biol..

[55] Hara T., Namba H., Yang T.T., Nagayama Y., Fukata S., Kuma K., Ishikawa N., Ito K., Yamashita S. (1998). Ionizing radiation activates c-Jun NH2-terminal kinase (JNK/SAPK) via a PKC-dependent pathway in human thyroid cells. Biochem. Biophys. Res. Commun..

[56] Toulany M., Kehlbach R., Florczak U., Sak A., Wang S., Chen J., Lobrich M., Rodemann H.P. (2008). Targeting of AKT1 enhances radiation toxicity of human tumor cells by inhibiting DNA-PKcs-dependent DNA double-strand break repair. Mol. Cancer Ther..

[57] Chou C.H., Chen S.U., Cheng J.C. (2009). Radiation-induced interleukin-6 expression through MAPK/p38/NF-kappaB signaling pathway and the resultant antiapoptotic effect on endothelial cells through Mcl-1 expression with sIL6-Ralpha. Int. J. Radiat. Oncol. Biol. Phys..

[58] Gao C., Kozlowska A., Nechaev S., Li H., Zhang Q., Hossain D.M., Kowolik C.M., Chu P., Swiderski P., Diamond D.J. (2013). TLR9 signaling in the tumor microenvironment initiates cancer recurrence after radiotherapy. Cancer Res..

[59] Itoh K., Wakabayashi N., Katoh Y., Ishii T., Igarashi K., Engel J.D., Yamamoto M. (1999). Keap1 represses nuclear activation of antioxidant responsive elements by Nrf2 through binding to the amino-terminal Neh2 domain. Genes. Dev..

[60] Zhang D.D., Lo S.C., Cross J.V., Templeton D.J., Hannink M. (2004). Keap1 is a redox-regulated substrate adaptor protein for a Cul3-dependent ubiquitin ligase complex. Mol. Cell. Biol..

[61] McDonald J.T., Kim K., Norris A.J., Vlashi E., Phillips T.M., Lagadec C., Della Donna L., Ratikan J., Szelag H., Hlatky L. (2010). Ionizing radiation activates the Nrf2 antioxidant response. Cancer Res..

[62] Feringa F.M., Raaijmakers J.A., Hadders M.A., Vaarting C., Macurek L., Heitink L., Krenning L., Medema R.H. (2018). Persistent repair intermediates induce senescence. Nat. Commun..

[63] Di Leonardo A., Linke S.P., Clarkin K., Wahl G.M. (1994). DNA damage triggers a prolonged p53-dependent G1 arrest and long-term induction of Cip1 in normal human fibroblasts. Genes Dev..

[64] Ianzini F., Mackey M.A. (1997). Spontaneous premature chromosome condensation and mitotic catastrophe following irradiation of HeLa S3 cells. Int. J. Radiat. Biol..

[65] Okayasu R., Okada M., Okabe A., Noguchi M., Takakura K., Takahashi S. (2006). Repair of DNA damage induced by accelerated heavy ions in mammalian cells proficient and deficient in the non-homologous end-joining pathway. Radiat. Res..

[66] Yim J.H., Yun J.M., Kim J.Y., Lee I.K., Nam S.Y., Kim C.S. (2017). Phosphoprotein profiles of candidate markers for early cellular responses to low-dose γ-radiation in normal human fibroblast cells. J. Radiat. Res..

[67] Mirzayans R., Scott A., Cameron M., Murray D. (2005). Induction of accelerated senescence by gamma radiation in human solid tumor-derived cell lines expressing wild-type TP53. Radiat. Res..

[68] Lowe S.W., Schmitt E.M., Smith S.W., Osborne B.A., Jacks T. (1993). p53 is required for radiation-induced apoptosis in mouse thymocytes. Nature.

[69] Strasser A., Harris A.W., Jacks T., Cory S. (1994). DNA damage can induce apoptosis in proliferating lymphoid cells via p53-independent mechanisms inhibitable by Bcl-2. Cell.

[70] Taneja N., Tjalkens R., Philbert M.A., Rehemtulla A. (2001). Irradiation of mitochondria initiates apoptosis in a cell free system. Oncogene.

[71] Liao E.C., Hsu Y.T., Chuah Q.Y., Lee Y.J., Hu J.Y., Huang T.C., Yang P.M., Chiu S.J. (2014). Radiation induces senescence and a bystander effect through metabolic alterations. Cell Death Dis..

[72] Coppé J.P., Patil C.K., Rodier F., Sun Y., Muñoz D.P., Goldstein J., Nelson P.S., Desprez P.Y., Campisi J. (2008). Senescence-associated secretory phenotypes reveal cell-nonautonomous functions of oncogenic RAS and the p53 tumor suppressor. PLoS Biol..

[73] Rodier F., Coppé J.P., Patil C.K., Hoeijmakers W.A., Muñoz D.P., Raza S.R., Freund A., Campeau E., Davalos A.R., Campisi J. (2009). Persistent DNA damage signalling triggers senescence-associated inflammatory cytokine secretion. Nat. Cell Biol..

[74] Wang Y., Zhao M., He S., Luo Y., Zhao Y., Cheng J., Gong Y., Xie J., Wang Y., Hu B. (2019). Necroptosis regulates tumor repopulation after radiotherapy via RIP1/RIP3/MLKL/JNK/IL8 pathway. J. Exp. Clin. Cancer Res..

[75] Cao W., Chen G., Wu L., Yu K.N., Sun M., Yang M., Jiang Y., Jiang Y., Xu Y., Peng S. (2023). Ionizing Radiation Triggers the Antitumor Immunity by Inducing Gasdermin E-Mediated Pyroptosis in Tumor Cells. Int. J. Radiat. Oncol. Biol. Phys..

[76] Ye L.F., Chaudhary K.R., Zandkarimi F., Harken A.D., Kinslow C.J., Upadhyayula P.S., Dovas A., Higgins D.M., Tan H., Zhang Y. (2020). Radiation-Induced Lipid Peroxidation Triggers Ferroptosis and Synergizes with Ferroptosis Inducers. ACS Chem. Biol..

[77] Paglin S., Hollister T., Delohery T., Hackett N., McMahill M., Sphicas E., Domingo D., Yahalom J. (2001). A novel response of cancer cells to radiation involves autophagy and formation of acidic vesicles. Cancer Res..

[78] Apel A., Herr I., Schwarz H., Rodemann H.P., Mayer A. (2008). Blocked autophagy sensitizes resistant carcinoma cells to radiation therapy. Cancer Res..

[79] Apetoh L., Ghiringhelli F., Tesniere A., Obeid M., Ortiz C., Criollo A., Mignot G., Maiuri M.C., Ullrich E., Saulnier P. (2007). Toll-like receptor 4-dependent contribution of the immune system to anticancer chemotherapy and radiotherapy. Nat. Med..

[80] Golden E.B., Frances D., Pellicciotta I., Demaria S., Helen Barcellos-Hoff M., Formenti S.C. (2014). Radiation fosters dose-dependent and chemotherapy-induced immunogenic cell death. Oncoimmunology.

[81] Obeid M., Panaretakis T., Joza N., Tufi R., Tesniere A., van Endert P., Zitvogel L., Kroemer G. (2007). Calreticulin exposure is required for the immunogenicity of gamma-irradiation and UVC light-induced apoptosis. Cell Death Differ..

[82] Chan Wah Hak C.M.L., Rullan A., Patin E.C., Pedersen M., Melcher A.A., Harrington K.J. (2022). Enhancing anti-tumour innate immunity by targeting the DNA damage response and pattern recognition receptors in combination with radiotherapy. Front. Oncol..

[83] Deng L., Liang H., Xu M., Yang X., Burnette B., Arina A., Li X.D., Mauceri H., Beckett M., Darga T. (2014). STING-Dependent Cytosolic DNA Sensing Promotes Radiation-Induced Type I Interferon-Dependent Antitumor Immunity in Immunogenic Tumors. Immunity.

[84] Zhu M., Yang M., Zhang J., Yin Y., Fan X., Zhang Y., Qin S., Zhang H., Yu F. (2021). Immunogenic Cell Death Induction by Ionizing Radiation. Front. Immunol..

[85] Vanpouille-Box C., Alard A., Aryankalayil M.J., Sarfraz Y., Diamond J.M., Schneider R.J., Inghirami G., Coleman C.N., Formenti S.C., Demaria S. (2017). DNA exonuclease Trex1 regulates radiotherapy-induced tumour immunogenicity. Nat. Commun..

[86] Baik J.S., Seo Y.N., Lee Y.C., Yi J.M., Rhee M.H., Park M.T., Kim S.D. (2022). Involvement of the p38 MAPK-NLRC4-Caspase-1 Pathway in Ionizing Radiation-Enhanced Macrophage IL-1β Production. Int. J. Mol. Sci..

[87] Mackenzie K.J., Carroll P., Martin C.A., Murina O., Fluteau A., Simpson D.J., Olova N., Sutcliffe H., Rainger J.K., Leitch A. (2017). cGAS surveillance of micronuclei links genome instability to innate immunity. Nature.

[88] Kerschbaum-Gruber S., Kleinwächter A., Popova K., Kneringer A., Appel L.M., Stasny K., Röhrer A., Dias A.B., Benedum J., Walch L. (2024). Cytosolic nucleic acid sensors and interferon beta-1 activation drive radiation-induced anti-tumour immune effects in human pancreatic cancer cells. Front. Immunol..

[89] Sun L., Wu J., Du F., Chen X., Chen Z.J. (2013). Cyclic GMP-AMP synthase is a cytosolic DNA sensor that activates the type I interferon pathway. Science.

[90] Withers S.S., Moeller C.E., Quick C.N., Liu C.C., Baham S.M., Looper J.S., Subramanian R., Kousoulas K.G. (2023). Effect of stimulator of interferon genes (STING) signaling on radiation-induced chemokine expression in human osteosarcoma cells. PLoS ONE.

[91] Obeid M., Tesniere A., Ghiringhelli F., Fimia G.M., Apetoh L., Perfettini J.L., Castedo M., Mignot G., Panaretakis T., Casares N. (2007). Calreticulin exposure dictates the immunogenicity of cancer cell death. Nat. Med..

